# Unusual manifestation of cystic mycetoma lesions: A case report

**DOI:** 10.1002/ccr3.8054

**Published:** 2023-10-17

**Authors:** Alaa Tajeldeen Habeeb Abdallah, Rami Elsiddig Abdelkhalig, Elwasila Hamid, Ayman Ahmed, Emmanuel Edwar Siddig

**Affiliations:** ^1^ Rufa'a Teaching Hospital Rufa Sudan; ^2^ General surgeon, Rufa'a Teaching Hospital Albutana University Rufa Sudan; ^3^ Consultant Surgeon, Rufa'a Teaching Hospital Albutana University Rufa Sudan; ^4^ Institute of Endemic diseases University of Khartoum Khartoum Sudan; ^5^ Swiss Tropical and Public Health Institute (Swiss TPH) Allschwil Switzerland; ^6^ University of Basel Basel Switzerland; ^7^ ErasmusMC, University Medical Center Rotterdam Department of Medical Microbiology and Infectious Diseases Rotterdam the Netherlands; ^8^ Faculty of Medical Laboratory sciences University of Khartoum Khartoum Sudan

**Keywords:** antibiotherapy, cystic presentation, mycetoma, sinuses and discharge, *Streptomyces somaliensis*

## Abstract

**Key Clinical Message:**

This case presents an atypical cystic presentation of mycetoma without sinuses or discharge. Awareness of these variations is crucial for accurate diagnosis and timely intervention, highlighting the need for healthcare professionals to consider diverse manifestations of mycetoma.

**Abstract:**

Mycetoma is a chronic and debilitating infectious disease characterized by localized swellings and granulomatous lesions. It primarily affects individuals in tropical and subtropical regions and is caused by certain fungi or bacteria. While mycetoma typically presents with sinuses and discharge, this case report presents a unique cystic presentation without these features. The patient, a 12‐year‐old female from Sudan, presented with a painless swelling on the dorsum of her right foot. Physical examination revealed a round, non‐tender, and fluctuant mass. Histopathological examination confirmed actinomycetoma caused by *Streptomyces somaliensis*. The patient was successfully treated with a combination of antibiotherapy. This atypical presentation underscores the need for healthcare professionals to consider uncommon variations of mycetoma for accurate diagnosis and management.

## Graphical abstract

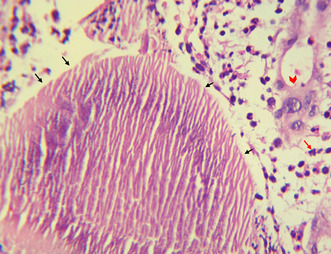

Graphical image shows the histopathological appearance of S. somaliensis grains (Black arrows) and surrounded by neutrophils (Red arrow) and epithelioid cells (red arrowhead) in H&E stained sections

## 1 INTRODUCTION

Mycetoma is a chronic and debilitating infectious disease characterized by the formation of localized swellings and granulomatous lesions in the skin, subcutaneous tissue, and sometimes even in bones.^1^ It primarily affects individuals residing in tropical and subtropical regions, where its prevalence is highest.^2^ This condition is caused by the invasion of certain fungi or bacteria into the body.^1^, ^2^

Mycetoma typically presents as painless, slow‐growing nodules or plaques that gradually enlarge over time.^3^ These lesions may initially appear as a small, firm bump and increase in size, eventually leading to disfigurement and functional impairment.^3^ The color of the skin overlying the affected area may change, becoming reddish, purplish, or brownish.^1^, ^3^

One of the key clinical features of mycetoma is the presence of sinuses or fistulas, which are deep tunnels that develop because of the inflammatory process.^3^ These sinuses often produce purulent, grain‐filled discharge, which may contain visible fungal or bacterial particles. The grains are identifiable as small, black, or colored structures containing the causative organisms.^4^

While mycetoma presents with distinctive clinical features, it shares similarities with other conditions, making a differential diagnosis crucial.^3^ Some of the differential diagnoses for mycetoma include bacterial abscesses,^5^ subcutaneous nodules of tuberculosis,^6^ syphilis gummas, neoplasms, and deep fungal infections.^3^

It is worth noting that while mycetoma is commonly associated with the formation of sinuses and fistulas, the occurrence of cystic lesions is rare. We present a unique case of mycetoma from Sudan that does not show the common characteristics of mycetoma infection.

## 2 CASE PRESENTATION

**FIGURE 1:**
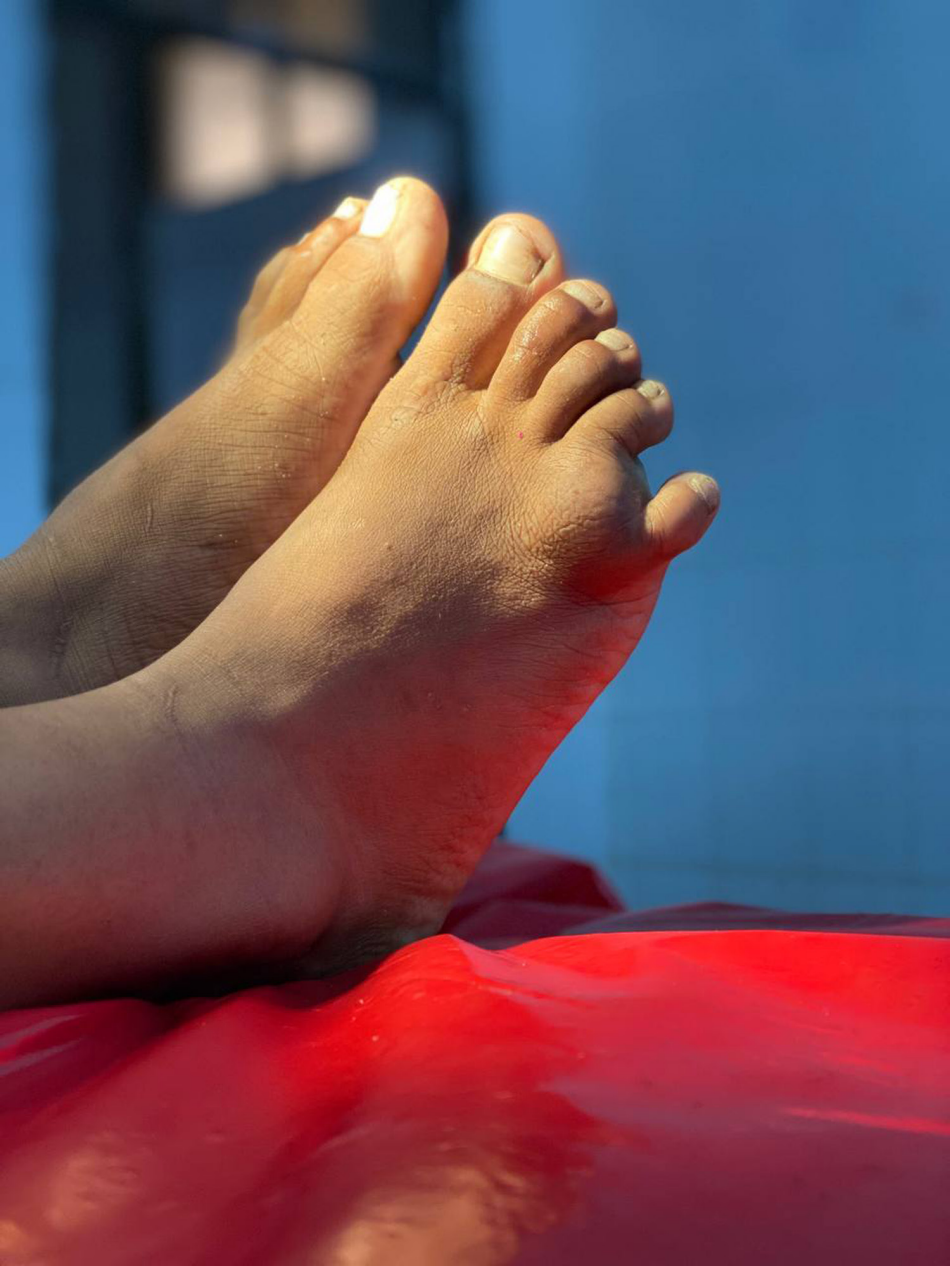
Photography showing the cystic lesion noticed at the dorsum of the foot.

A 12‐year‐old female student from Fao, Gadarif state, Sudan presented to Rufa'a Teaching Hospital with a complaint of swelling in her right foot for the past 5 months. The swelling was located on the dorsum of the foot, proximal to the fourth and fifth toes (Figure 1). It had been gradually increasing in size but was painless. There was no history of sinuses or discharge. The patient mentioned having a regular habit of going into pool water but denied any traumatic event. She had a history of allergic conjunctivitis but was otherwise healthy. There was no previous record of a similar condition or operation.

During the physical examination, a 4 × 2 cm round mass was identified on the dorsum of the right foot, involving the fourth and fifth toes. The skin over the mass appeared normal in shape and color, with no sinuses or discharges. The swelling was smooth, non‐tender on palpation, not attached to the skin, and showed mobility proximally but lesser mobility distally. It felt fluctuant, indicating a fluid content. The regional lymph nodes were unaffected.

Her complete blood count examinations reported normal total white blood cell count (WBCs) of 7.9 × 10^3^, hemoglobin count of 16.9 g/dL, and platelet count of 225 × 10^3^. Viral screening for human immunodeficiency virus (HIV), Hepatitis B and C all were negative.

**FIGURE 2:**
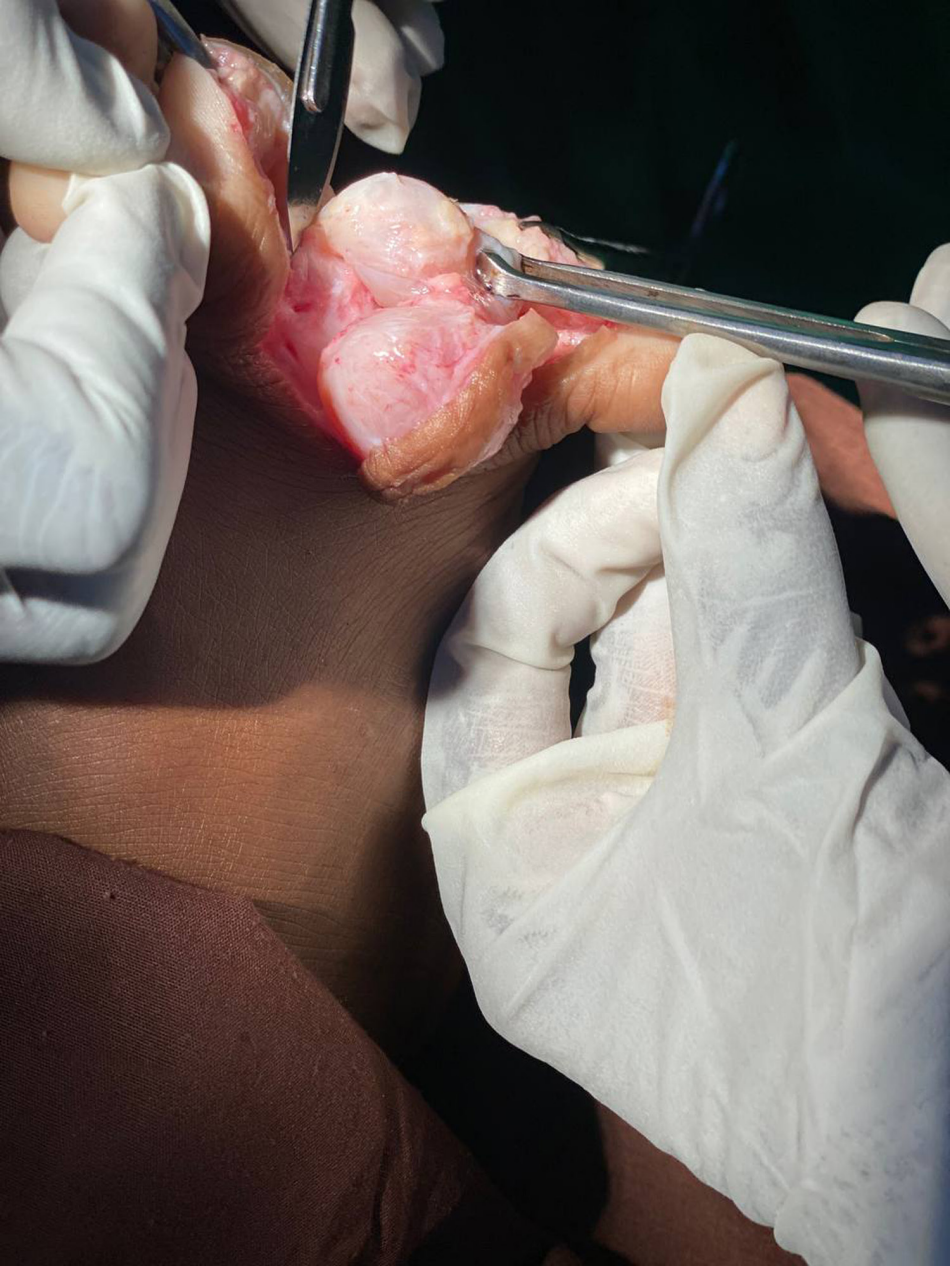
Photography showing the presence of two cyst.

Due to the findings, the patient was prepared for surgical intervention under general anesthesia. A tourniquet was applied to maintain a bloodless field, and an interdigital incision was made from the dorsum of the foot to the plantar side between the fourth and fifth toes. The layers of the skin were dissected, revealing a round cyst. Slow dissection of the cyst edges was performed to prevent rupture. Another cyst was discovered at the base of the first cyst, extending medially to the plantar side of the fourth toe (Figure 2). Both cysts were completely excised, and the skin was sutured. The excised lesions were sent to the histopathology laboratory and immersed in 10% neutral buffered formalin for further examination.

**FIGURE 3:**
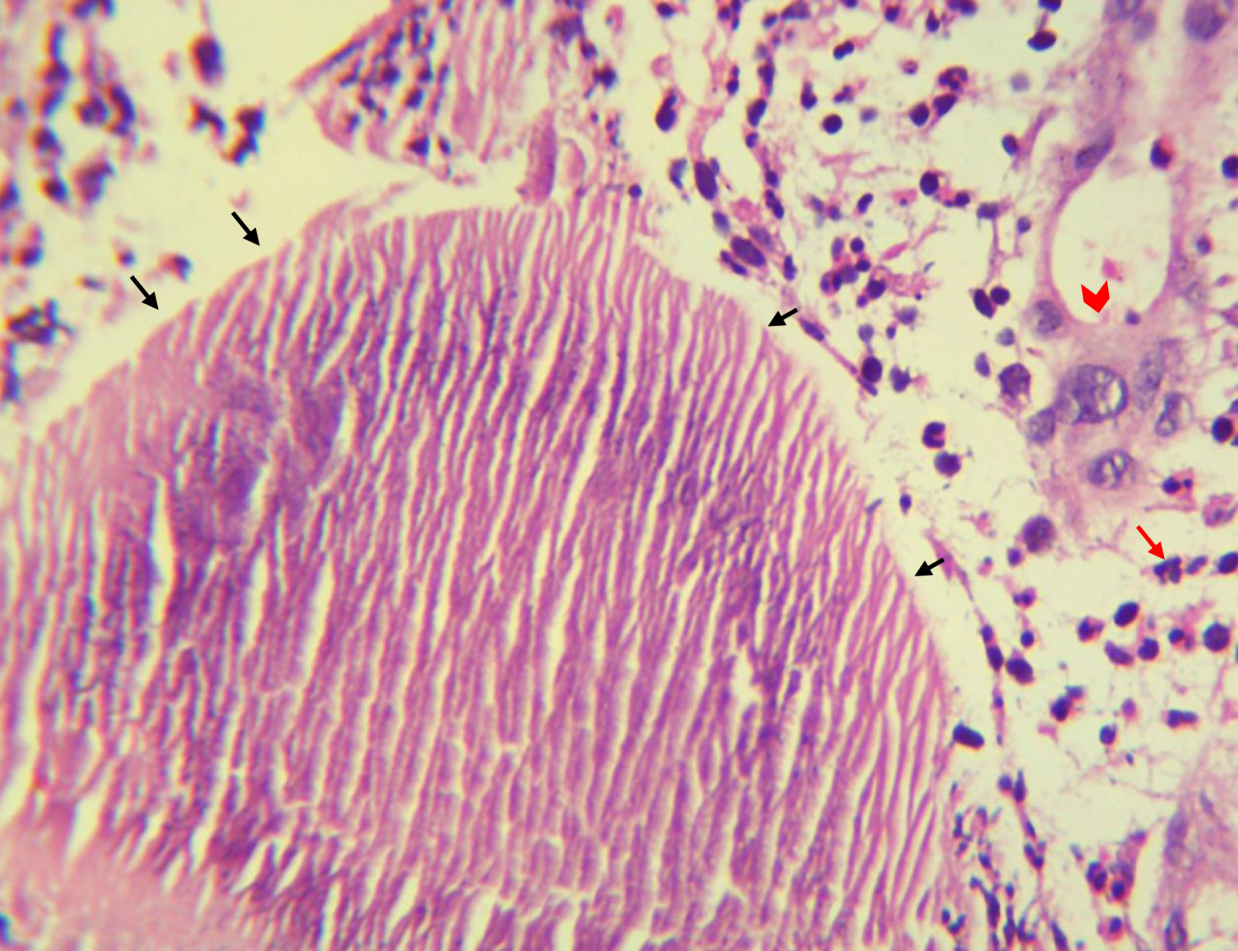
The histopathological appearance of *Streptomyces somaliensis* grains (black arrows) and surrounded by neutrophils (red arrow) and epithelioid cells (red arrowhead) in H&E stained sections (Magnification 40×).

In the histopathology laboratory, the cyst was opened, revealing white to yellow materials that were small, tiny in size, and soft in consistency. The histopathological examination of the surgical biopsy taken from the cyst showed scattered grains. In the sections, the bacteria were surrounded by a zone of a large eosinophilic matrix with club‐like projections. The grains had cracks, and the bacteria stained faint pink with Hematoxylin and Eosin (H&E). Based on histology a *Streptomyces somaliensis* actinomycetoma was established (Figure 3). The patient received streptomycin 25–30 mg/kg IM 2 times/week for 1 week; and amoxicillin/clavulanic acid (Amoclan) 625 mg twice per day with favorable outcome after 2 months from the surgery.

## 3 DISCUSSION

The presented case highlights a unique presentation of mycetoma in a 12‐year‐old female student from Fao, Gadarif state, Sudan. Unlike the typical mycetoma clinical features,^2^, ^7^, ^8^ this case exhibited a cystic presentation without the presence of sinuses or discharge. The patient's history indicated a gradual increase in swelling over the past 5 months, located on the dorsum of the right foot, proximal to the fourth and fifth toes. The swelling was painless and not associated with any traumatic event.

Mycetoma is a chronic infectious disease characterized by the formation of granulomas, primarily affecting the extremities.^2^, ^9^ However, it has also been reported in other parts of the body.^2^ The disease can be caused by either bacteria or fungi, known as actinomycetoma and eumycetoma, respectively.^2^ The clinical suspicion of mycetoma is typically based on a triad of symptoms, including progressive painless swelling beneath the skin, the formation of sinus tracts, and discharge with a granular appearance.^7^, ^8^ However, in the case of our patient, they presented with swelling alone, without the presence of sinuses or discharge.

Mycetoma primarily occurs in rural areas and it is more commonly observed among male laborers who work without footwear.^2^ It predominantly affects the foot, accounting for approximately 70% of cases, followed by the hand (15%).^2^ Interestingly, in our specific case, the patient had a habit of regularly immersing themselves in water, which may have contributed to the development of mycetoma. Additionally, it is worth noting that our patient was relatively young, only 12 years old, which is considered one of the age groups most affected by mycetoma.^2^

The physical examination revealed a round, smooth, non‐tender, and fluctuant mass. The absence of attachment to the skin and increased mobility proximally supported the cystic nature of the lesion. Cystic mycetoma lesion is rarely encountered, furthermore in the study conducted by Bonifaz et al., out of the 482 cases examined, only two cases (0.41%) presented with a cystic formation, while the majority of cases (97%) showed tumor‐like appearances accompanied by draining sinuses.^10^ The fluid found within the cysts in these cases was identified as exudate, which resulted from leakage from the thick capsules lining the granulation tissue.

Accurate diagnosis is crucial for proper patient management, especially when it comes to identifying the causative organism and providing appropriate treatment.^11^ In the case we are discussing, the patient was diagnosed with actinomycetoma caused by *Streptomyces somaliensis* through histopathological examination. In order to identify the causative agents, various diagnostic tools were employed. Culture techniques were used to isolate and grow the microorganism in a laboratory setting.^4^ Fine needle aspiration cytology (FNAC) was utilized to collect samples from the affected area for microscopic examination and assessment.^4^, ^12^

Treatment for *Streptomyces somaliensis* actinomycetoma typically involves a combination of antibiotherapy, including streptomycin and co‐trimoxazole, which was successful in this case. The favorable outcome suggests the effectiveness of this treatment regimen.

Unfortunately, in the limited resources setting of Sudan that is endemic with several infectious diseases commonly manifested with similar clinical symptoms, misdiagnosis or delay in reaching the final diagnosis are increasingly reported in Sudan.^13^ The atypical manifestation, severe prognosis of infection, and co‐infections are adding more challenge for healthcare providers and the quality of their healthcare.^14^, ^15^, ^16^, ^17^, ^18^, ^19^ This atypical presentation of infectious diseases including mycetoma highlights the need for improving the diagnostic capacity particularly in areas endemic with more than one of infectious diseases with overlapping symptoms.^6^, ^20^, ^21^ This improvement needs to be at the education and continuous training of healthcare providers as well as the infrastructure and integrating the use of more robust diagnostic tools such as molecular and genomic sequencing in these endemic areas.^12^ Healthcare providers in such settings should be highly skeptical and try to thoroughly examine their patients and carefully revising their travel and medical history. However, this needs to be done cost‐effectively and considerately to avoid increasing the health and socioeconomic burden over the patient.

However, in other settings where mycetoma is not as common, it can present as a mimic of various other conditions. It may resemble tuberculosis, osteomyelitis (inflammation of the bone marrow), other fungal infections, as well as soft tissue tumors. This similarity in presentation can make the diagnosis of mycetoma more challenging since it may be easily mistaken for these other conditions. Therefore, in areas where mycetoma is not endemic, it is crucial for healthcare professionals to consider mycetoma as a potential differential diagnosis when encountering subcutaneous masses, especially if the patient has a history of travel to or residence in endemic regions. This ensures that appropriate investigations and management options were explored to correctly identify and treat mycetoma cases.

## AUTHOR CONTRIBUTIONS

**Alaa Tajeldeen Habeeb Abdallah:** Conceptualization; data curation; formal analysis; funding acquisition; investigation; methodology; project administration; supervision; validation; visualization; writing – review and editing. **Rami Elsiddig Abdelkhalig:** Conceptualization; data curation; formal analysis; funding acquisition; investigation; methodology; project administration; supervision; validation; visualization; writing – review and editing. **Elwasila Hamid:** Conceptualization; data curation; formal analysis; funding acquisition; investigation; methodology; project administration; validation; visualization; writing – review and editing. **Ayman Ahmed:** Conceptualization; data curation; formal analysis; funding acquisition; investigation; methodology; project administration; supervision; validation; visualization; writing – original draft; writing – review and editing. **Emmanuel Edwar Siddig:** Conceptualization; data curation; formal analysis; funding acquisition; investigation; methodology; project administration; supervision; validation; visualization; writing – original draft; writing – review and editing.

## FUNDING INFORMATION

None.

## CONFLICT OF INTEREST STATEMENT

The author reports no conflicts of interest in this work.

## CONSENT

A written consent form was obtained from patient Legal Guardian.

## Data Availability

The data that support the findings of this study are available from the corresponding author upon reasonable request.

## References

[1] Seas C , Legua P . Mycetoma, chromoblastomycosis and other deep fungal infections: diagnostic and treatment approach. Curr Opin Infect Dis. 2022;35(5):379‐383. 35942857 10.1097/QCO.0000000000000870

[2] van de Sande WW . Global burden of human mycetoma: a systematic review and meta‐analysis. PLoS Negl Trop Dis. 2013;7(11):e2550. 24244780 10.1371/journal.pntd.0002550 PMC3820768

[3] Bawaskar HS , Bawaskar PH . Mycetoma. J Assoc Physicians India. 2021;69(11):11‐12. 34781618

[4] Siddig EE , Mhmoud NA , Bakhiet SM , et al. The accuracy of histopathological and cytopathological techniques in the identification of the mycetoma causative agents. PLoS Negl Trop Dis. 2019;13(8):e0007056. 31465459 10.1371/journal.pntd.0007056 PMC6750607

[5] Siddig EE , Nyuykonge B , Bakheit OEH , et al. Staphylococcus aureus causing primary foot botryomycosis mimicking actinomycetoma: a case report from Sudan. Int J Infect Dis. 2022;124:224‐226. 36241164 10.1016/j.ijid.2022.10.010

[6] Ahmed A , Hagelnur AA , Eltigani HF , Siddig EE . Cutaneous tuberculosis of the foot clinically mimicking mycetoma: a case report. Clin Case Rep. 2023;11(5):e7295. 37151934 10.1002/ccr3.7295 PMC10160425

[7] Emmanuel P , Dumre SP , John S , Karbwang J , Hirayama K . Mycetoma: a clinical dilemma in resource limited settings. Ann Clin Microbiol Antimicrob. 2018;17(1):35. 30097030 10.1186/s12941-018-0287-4 PMC6085652

[8] Agarwal P , Jagati A , Rathod SP , Kalra K , Patel S , Chaudhari M . Clinical features of mycetoma and the appropriate treatment options. Res Rep Trop Med. 2021;12:173‐179. 34267575 10.2147/RRTM.S282266 PMC8275212

[9] Bellalah A , Abdeljelil NB , Njima M , et al. Cystic form of Actinomycotic mycetoma: a new case with a diagnostic challenge. Clin Case Rep. 2021;9(4):e04064. 33936735 10.1002/ccr3.4064 PMC8077338

[10] Bonifaz A , Tirado‐Sánchez A , Calderón L , et al. Mycetoma: experience of 482 cases in a single center in Mexico. PLoS Negl Trop Dis. 2014;21(8):e3102. 10.1371/journal.pntd.0003102 PMC4140667 25144462

[11] Siddig EE , Ahmed A , Ali Y , et al. Eumycetoma medical treatment: past, current practice, latest advances and perspectives. Microbiol Res. 2021;12(4):899‐906.

[12] Siddig EE , Ahmed A , Hassan OB , et al. Using a Madurella mycetomatis‐specific PCR on grains obtained via non‐invasive fine‐needle aspirated material is more accurate than cytology. Mycoses. 2023;66(6):477‐482. 36740735 10.1111/myc.13572

[13] Ahmed A , Eldigail M , Elduma A , et al. First report of epidemic dengue fever and malaria co‐infections among internally displaced persons in humanitarian camps of North Darfur. Sudan Int J Infect Dis. 2021;108:513‐516. 34044142 10.1016/j.ijid.2021.05.052 PMC8860570

[14] Siddig EE , Ahmed A . When parasites stray from the path: a curious case of ectopic cutaneous Schistosoma haematobium. QJM. 2023;31:794‐795. doi:10.1093/qjmed/hcad112 37255318

[15] Mohamed AK , Elhassan NM , Awhag ZA , et al. Prevalence of helicobacter pylori among Sudanese patients diagnosed with colon polyps and colon cancer using immunohistochemistry technique. BMC Res Notes. 2020;13(1):322. 32631443 10.1186/s13104-020-05159-2 PMC7339555

[16] Ahmed A , El‐Amin R , Musa AM , et al. Guillain‐Barre syndrome associated with COVID‐19 infection: a case series. Clin Case Rep. 2023;11:e6988. 36852114 10.1002/ccr3.6988 PMC9957700

[17] Ali Y , Siddig E , Mohamed N , Ahmed A . Rift valley fever (RVF) and malaria co‐infection: a case report. Clin Case Rep. 2023;11(9):e7926. 37731970 10.1002/ccr3.7926 PMC10507219

[18] Ahmed A , El‐Sadig SM , Eltigani H , Siddig E . Guillain‐Barre syndrome associated with COVID‐19 and malaria coinfection: a case report. Authorea. 2023. doi:10.22541/au.167767321.12356900/v1

[19] Ahmed A , El‐Sadig SM , Siddig EE . Guillain‐Barre syndrome associated with hepatitis E virus infection: a case report. Clin Case Rep. 2023;11(9):e7863. 37655129 10.1002/ccr3.7863 PMC10465721

[20] Siddig EE , Ahmed A , Eltigani HF , Bakhiet SM , van de Sande WWJ , Fahal AH . The first case of *Fusarium falciforme* eumycetoma in Sudan and an extensive literature review about treatment worldwide. J Fungi (Basel). 2023;9(7):730. 37504719 10.3390/jof9070730 PMC10381130

[21] Ahmed A , Hemaida MA , Hagelnur AA , Eltigani HF , Siddig EE . Sudden emergence and spread of cutaneous larva migrans in Sudan: a case series calls for urgent actions. IDCases. 2023;32:e01789. 37207175 10.1016/j.idcr.2023.e01789 PMC10189479

